# Usability Evaluation Ecological Validity: Is More Always Better?

**DOI:** 10.3390/healthcare12141417

**Published:** 2024-07-16

**Authors:** Romaric Marcilly, Helen Monkman, Sylvia Pelayo, Blake J. Lesselroth

**Affiliations:** 1 Univ. Lille, CHU Lille, ULR 2694—METRICS: Évaluation des Technologies de Santé et des Pratiques Médicales, F-59000 Lille, France; sylvia.pelayo@univ-lille.fr; 2 Inserm, CIC-IT 1403, F-59000 Lille, France; 3 School of Health Information Science, University of Victoria, Victoria, BC V8P 5C2, Canada; monkman@uvic.ca (H.M.); blake-lesselroth@ouhsc.edu (B.J.L.); 4 School of Community Medicine, University of Oklahoma, Tulsa, OK 74135, USA

**Keywords:** usability, evaluation, user testing, ecological validity, nociception index

## Abstract

Background: The ecological validity associated with usability testing of health information technologies (HITs) can affect test results and the predictability of real-world performance. It is, therefore, necessary to identify conditions with the greatest effect on validity. Method: We conducted a comparative analysis of two usability testing conditions. We tested a HIT designed for anesthesiologists to detect pain signals and compared two fidelity levels of ecological validity. We measured the difference in the number and type of use errors identified between high and low-fidelity experimental conditions. Results: We identified the same error types in both test conditions, although the number of errors varied as a function of the condition. The difference in total error counts was relatively modest and not consistent across levels of severity. Conclusions: Increasing ecological validity does not invariably increase the ability to detect use errors. Our findings suggest that low-fidelity tests are an efficient way to identify and mitigate usability issues affecting ease of use, effectiveness, and safety. We believe early low-fidelity testing is an efficient but underused way to maximize the value of usability testing.

## 1. Introduction

Ecological validity (i.e., “test fidelity”) is “*the extent to which behaviour in a test situation can be generalised to a natural setting*” [1]. Many testing conditions, such as the realism of the physical environment or the responsiveness of software functionality, can affect test validity and the ability to predict real-world performance. Ecological validity is high when each test condition closely mimics reality [2], whereas differences between the live and test environments may limit the accuracy of observations [3,4,5,6]. Borycki and Kushniruk’s Cognitive Socio-Technical Framework [7] says ecological validity should increase as tests move from the bench to the bedside. However, this is not always possible because of organizational resource constraints, including time, money, and the availability of skilled testers. It is, therefore, important to identify which conditions have the greatest effect on validity and estimate the cost-effectiveness of replicating them with the highest possible fidelity.

Studies have investigated the effect of ecological validity on usability testing outcomes. Most of them evaluated only one ecological validity dimension at a time, usually comparing two levels of that dimension [8,9,10]. Only a few studies have looked at multiple dimensions concurrently [3,4]. Our goal was to study how manipulating several dimensions of ecological validity simultaneously can affect testing results, including detecting technology use errors [8]. We focused on summative testing of an acute care and intraoperative pain monitor. We compared two levels of ecological fidelity and asked the following questions: (1) were all use errors detected in both test settings? (2) did the level of fidelity influence the number of errors made by participants? (3) were any novel errors (i.e., not identified in the risk analysis) detected?

## 2. Models of Ecological Validity

Many research teams have published models of ecological validity [3,11]. For example, Sauer’s Four-Factor Framework of Contextual Validity categorizes conditions according to activity system components (i.e., user, technology, task, and environment). For our study, we used van Berkel’s Seven Dimensions Framework (2020). This model lists seven dimensions of ecological validity that are important to consider when designing usability studies: (1) user roles, (2) the evaluation environment, (3) the presence of user training, (4) the clinical scenario, (5) whether patients are involved during testing, (6) attributes of the hardware, and (7) the software. We chose this model because it captured the largest number of variables in the most granular detail across the technology development lifecycle. In the next section, we describe each dimension in greater detail [11].

### 2.1. User Roles

It is important to recruit representative end-users for testing whenever possible rather than developers or professional testers. People involved in the design process—including clinician developers—cannot substitute for actual users [11,12]. The characteristics of the participant sample, such as professional role (e.g., physician, nurse, specialist), technical skill (e.g., computer literacy, years of experience), level of clinical training, usage habits, and preferences or values about the task or technology should match the target population to ensure the sample is representative of intended end-users [3,4,6,11]. There is some disagreement among experts on whether it is important to recruit only novices [13] or novices and experts [3,14,15]. We believe the decision to recruit multiple levels of user expertise depends on the objectives of the test (e.g., counting the number of usability issues, estimating learnability, or measuring error tolerance) [3]. Participants’ conditions during testing (e.g., fatigue, beginning vs. end of shift, mood) may also affect test results and should match the end-user’s context during actual use. To limit inequity or implicit bias, it may also be necessary to account for age, gender, and ethnicity.

While including representative end-users in testing is always preferable, it is often challenging to recruit healthcare professionals with the necessary subject matter expertise, knowledge of the target setting, and who have protected time to participate in testing. Usability professionals often must adapt and improvise to meet sponsor deadlines. If recruiting all representative users is cost or time-prohibitive, we suggest developing user personas. Personas are fictional but evidence-based representations of user groups that can guide recruitment strategies or testers [16]. We believe personas are most helpful for identifying technology requirements and guiding early design decisions but should not replace testing with representative users.

### 2.2. Environment

The environment dimension includes the physical (i.e., “*built*”) and the social environment. The physical test environment refers to attributes of the facility or equipment that influence user behavior. This might include the configuration of the testing room or the presence of background noise. Test environment fidelity could range from an administrative office equipped with a desktop computer (i.e., low fidelity) to a simulation lab reproduction of a hospital room (i.e., high fidelity) to an actual unoccupied hospital room (i.e., naturalistic) [6]. The social environment refers to the presence of other humans or workflow interference (e.g., competing clinical requests, text messages, and pages) during the test [1]. Some tasks require multiple healthcare providers to work collaboratively. Teamwork and interprofessional interactions can be reproduced using scripts and actions performed by members of the research team (i.e., low fidelity), actors (i.e., high fidelity), or real-life colleagues (i.e., naturalistic).

While replicating real-world conditions is desirable [2,3,4,6,11,17,18,19,20,21], especially during safety investigations [6,22], it is often not feasible given cost constraints. For example, testing a new surgical device a surgeon uses might call for a detailed simulation of the operating theatre, including medical equipment, an interactive mannequin, and actors to portray the interdisciplinary team. Unfortunately, many manufacturers and organizations cannot afford this level of ecological validity. In these circumstances, usability professionals must compromise between the realism of the test conditions and the built environment [23]. While it is important to replicate the most important attributes of the real environment, this is an unresolved area of active research [8,24,25,26,27,28,29,30,31].

### 2.3. Training

Van Berkel et al. [11] pointed out that researchers often train participants on a system before testing in a simulation. To avoid confounding, test proctors must offer the same training and materials actual users would receive. Including product training as part of the testing protocol can identify education gaps and ways to improve new user orientation [11].

### 2.4. Test Scenario

The test scenario provides context for the test participants; it describes the clinical use case, care setting, and task goals [32]. The fidelity of the scenario influences how seriously participants behave in a study (i.e., behavioral fidelity) [11]. In goal-based scenarios, usability professionals observe participants as they determine and execute the steps necessary to achieve the goal [32]. These are higher fidelity than simple tests of product features or user acceptance tests wherein proctors provide participants with step-by-step instructions to complete tasks. The breadth and depth of scenarios should, therefore, be representative of real-life activities [3]. Breadth is the extent to which activity system complexity is captured in the test (e.g., single task vs. parallel tasks) [3]. Depth is the level of detail and completeness with which a task is simulated (i.e., the proportion of real-life steps included in the test) [3,6]. The instructions researchers give testers for reporting findings can influence the results [18,19,20]. For example, with static prototypes (e.g., drawings, wireframes), participants may be asked to describe what they would do (e.g., click, type, scroll) to operate a product, whereas with interactive prototypes, participants may verbalize their thoughts while using the product.

Kushniruk and colleagues also suggested explicitly defining the urgency and typicality of tasks when designing the scenario [6]. Urgency indicates the level of immediacy and pressure associated with completing a task. Tasks can range from non-urgent (e.g., submitting a routine electronic order for acetaminophen) to urgent (e.g., submitting an order for a stat antihypertensive during a medical emergency). Typicality refers to how a task represents the usual, normal, or expected system use or workflow. Both extremes (i.e., typical and atypical) can be important during testing. For example, minor—yet common—usability issues may profoundly affect efficiency and user satisfaction. Rare usability issues may be more difficult to detect and cause catastrophic outcomes [12].

### 2.5. Patient Involvement

Van Berkel et al. (2020) [11] cautioned that including real patients in usability testing can generate valuable insights but at considerable risk. Patients may identify usability issues that are impossible to detect with actors. However, there are potential patient safety risks. There are both physical and psychological risks to consider. For example, a patient participating in an interview may re-experience painful events or memories. Usability testing, therefore, often includes a proxy for patients. For example, a study might instead use a mannequin or actor (i.e., a standardized patient) [8].

### 2.6. Software

Usability professionals sometimes conduct tests using early HIT prototypes (e.g., paper prototypes or wireframes). Generally, the prototyping method and degree of realism can influence participants’ reactions. The dimensions to consider include feature breadth (i.e., the proportion of finished features present), feature depth (i.e., level of feature detail) [3], physical similarity, interaction similarity, visual appearance [33], and data similarity [11].

While it is important to test as early as possible in the product design lifecycle—even with paper prototypes—there is a complex interaction between prototype fidelity and outcome measures [3,5,9,34,35]. Some studies suggest that prototype fidelity does not affect the number and type of usability problems detected [3,9]. However, we believe that when measuring participant behavior (e.g., clinically relevant performance), efficiency (e.g., task completion time), and effectiveness (e.g., task success rate), prototype fidelity is relevant [5,34,35]. Furthermore, it may be necessary to pre-populate the system with patient data. These data might be fabricated or real, anonymized patient data. If fabricated, it is important to include extreme values and test at the edges of input ranges (i.e., “boundary value analysis”) to identify rare occurrences.

### 2.7. Hardware

Technology hardware can create usability issues or affect the goal success rate. A study by Andre et al. looked at the design and performance of four automatic external defibrillators [36]. The team found that participants could not use two machines to deliver a shock. The hardware design and packaging significantly influenced the ability of untrained caregivers to use the equipment properly. While hardware should be accounted for when designing tests or evaluating usability data, high-fidelity hardware prototypes are often expensive and time-consuming to produce [6].

## 3. Materials and Methods

### 3.1. Health Information Technology

We studied a novel pain monitor that uses calculations from an electrocardiogram (ECG) tracing to estimate the autonomic nervous system response to painful or stressful stimuli. The HIT measures the R-R interval between two QRS complexes [37]. An algorithm then calculates an analgesia nociception index (ANI): a unitless index ranging between 0 and 100, with higher values indicating more parasympathetic activity associated with analgesia and lower values indicating more sympathetic activity associated with pain (i.e., 0 = great pain or stress; 100 = adequate anesthesia). The goal is to keep the patient’s ANI between 50–70.

**Figure 1:**
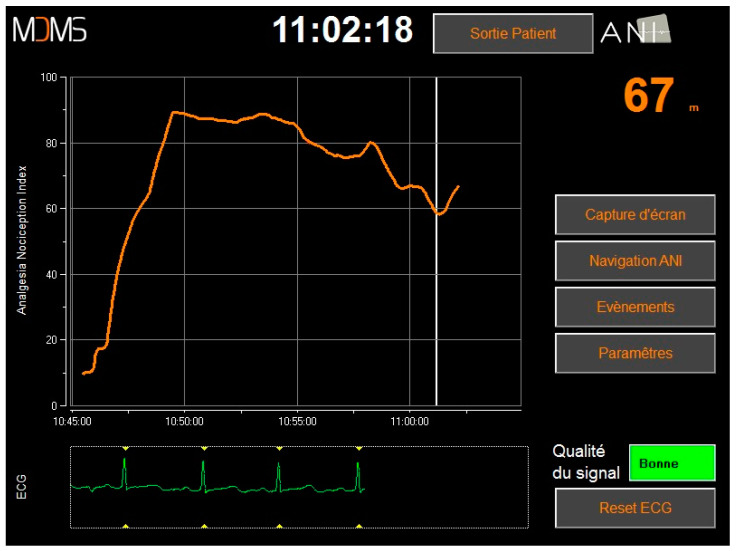
Screenshot of the analgesia nociception index (ANI) monitor.

The graphical user interface of the HIT (Figure 1) displays the instantaneous value of the ANI (ANIi) and its average over time (mean ANI, ANIm). The display includes numerical values, graphs, and information about the ECG and signal quality. The ANI monitor must be reset between patients to avoid errors (i.e., new patient data displayed with the previous patient’s threshold calibration). The pain monitor can be used in intensive care units, operative theatres, and post-surgical care units. It is typically located at the head of the bed, close to other vital sign monitors.

### 3.2. Risk Analysis to Inform Scenario Development

We used a mixture of published articles and grey literature to conduct an a priori risk analysis. We studied safety incident reports databases and complaints files, published usability studies on earlier versions of the pain monitor, and conducted interviews with end-users using similar devices to identify potential usability errors and their consequences. We identified eight usability errors (Table 1) associated with physician and nursing tasks. We classified errors into three severity levels: mild: no injury, no patient discomfort (*n* = 1 error); moderate: light patient discomfort (*n* = 4 errors); severe: serious injury or death (*n* = 3 errors). We developed scenarios to test for each of the eight use errors.

### 3.3. Participants

In France, physicians and nurses with specialized training in anesthesiology typically manage pain in dedicated anesthesiology and resuscitation units. We conducted a preliminary context-of-use analysis in these two units and found no differences in how the devices were used. Therefore, to gather comprehensive data on the types of errors encountered with the ANI monitor, we recruited physicians and nurses for this study.

We included participants in this study if they were: (1) physicians (i.e., resuscitation clinicians or anesthesiologists) or nurses specialized in intensive care, (2) had at least two months of professional experience in an intensive care unit, (3) completed training with the ANI monitor, and (4) consented to be recorded. Participants were excluded if they had previously used this ANI pain monitor. Recruitment proceeded through convenience sampling. We recruited volunteers through announcements (i.e., newsletters and emails) in Lille Academic Hospital’s units and through their professional networks.

### 3.4. Study Design and Test Conditions

**Figure 2:**
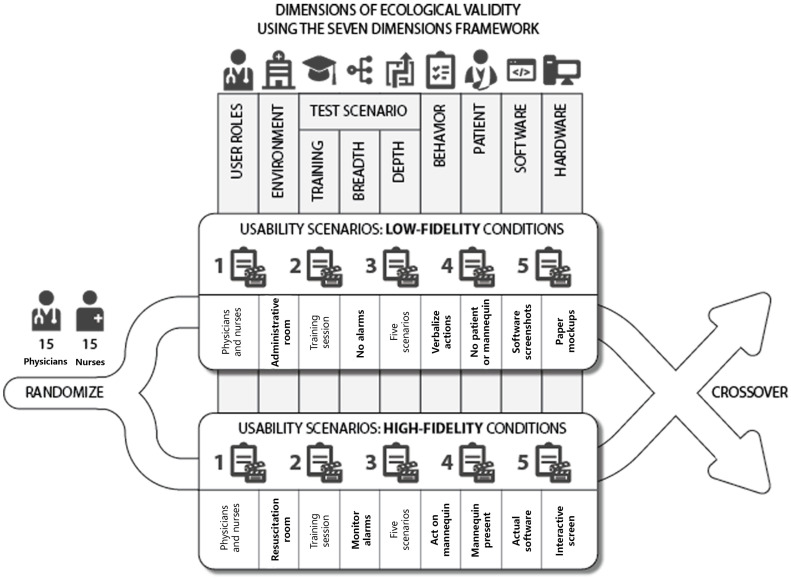
Diagram of the within-subjects study design. Thirty physicians and nurses completed five low-fidelity scenarios and five high-fidelity scenarios. We changed the fidelity in six of the seven ecological validity dimensions.

We performed an in-lab experimental study using a one-factor within-subjects design (Figure 2). The within-participants variable was the level of fidelity; this included two conditions: low fidelity and high fidelity. While ecological validity can be conceptualized on a continuum, we designed two discrete levels for our study: low-fidelity and high-fidelity. We manipulated multiple ecological dimensions for each level (i.e., environment, scenario, patient involvement, software) (Table 2). All participants completed the same five scenarios twice—once for each test condition (i.e., low- and high-fidelity). We furnished each subject with two different but equivalent clinical cases to limit any carryover or priming effect. We counterbalanced the exposure order for each condition and clinical case. All test scenarios were developed by an anesthesiologist and modeled after real patient cases. A second anesthesiologist reviewed each case for face and content validity. During testing, a proctor observed participants either through one-way glass (high-fidelity) or while standing in the same room (low-fidelity). The proctor gave instructions through a loudspeaker (high-fidelity) or in person (low-fidelity). We audio- and video-recorded all usability tests.

### 3.5. Measurements

We recorded use errors during each test. We first interviewed two anesthesiologists and created a reference standard of acceptable behaviors and answers for each scenario. In the high-fidelity condition, we asked participants to behave in each scenario as they would in real life. In the low-fidelity condition, we instructed them to tell the observer what they would do for each task. During testing, a usability professional compared test subject behaviors and answers to the reference standard. For double-pass verification, a second usability professional reviewed all recordings and scored behaviors or answers.

### 3.6. Procedures

After determining the eligibility and consent of each participant, we explained the testing procedure. Then, participants completed all five scenarios in one condition (i.e., either high- or low-fidelity) and, after a short break, completed the next five scenarios in the other condition. We determined the order of scenarios in each condition using a randomization table. After the tests, we held debriefing sessions using a semi-structured interview guide to explore participants’ perspectives on the technology (e.g., perceived usefulness and usability) and the root cause(s) of their errors. The total test duration was approximately 70 min.

### 3.7. Statistical Analyses

For each test and each participant, we counted the total number of errors and scored each by type and severity (i.e., mild, moderate, severe). We calculated descriptive statistics for errors at each level of fidelity. We calculated the overall frequency of error occurrence and the frequency by ecological level. Due to the small sample size and the rarity of the errors, we could not calculate inferential statistics. Statistical analyses were performed with Jamovi software (version 2.3.21, The Jamovi project).

### 3.8. Ethical Considerations

This study was conducted in France and is categorized as human and social science research. In accordance with French biomedical research law, our study protocol was exempt from ethical board approval or oversight [38,39]. This study was conducted in accordance with the Declaration of Helsinki.

We recruited all participants voluntarily and provided financial compensation of 150€ (approximately 160 U.S.$) for participation.

## 4. Results

Both groups were similar in age and sex (Table 3).

**Figure 3:**
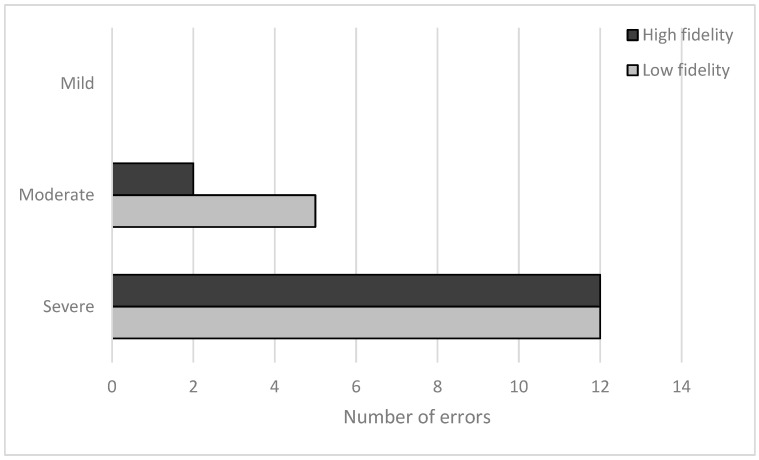
Number of errors committed in each condition according to the severity of the errors.

Across both conditions, we identified thirty-one errors (Figure 3); there were seven moderate and twenty-four severe errors. We saw users commit five of the eight possible error types (Figure 3 and Table 4) listed in our risk analysis (Table 1). Four error types appeared in both conditions (#2, #6, #7, and #8), and one only appeared in the low-fidelity condition (#4). We did not identify any unexpected errors (i.e., errors that defied categorization according to our risk analysis) in either condition.

Across both conditions, participants made, on average, 1.03 errors (range = 0–7) out of 16 possible errors (8 errors × 2 conditions). They made 0.57 (range = 0–4) out of 8 possible errors in the low-fidelity tests and 0.47 (range = 0–3) in the high-fidelity tests. Sixteen participants (53%) did not commit any errors in either condition. Three participants did not commit any errors in the low-fidelity condition (10%), and one participant did not commit any errors in the high-fidelity condition (3%).

**Figure 4:**
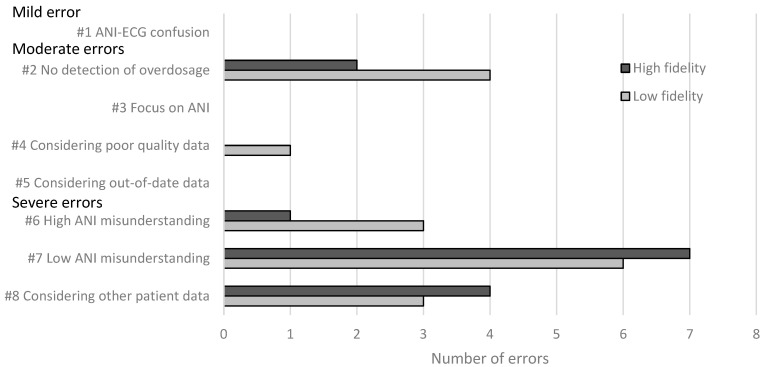
Number of errors made by the participants according to the type of error and the test condition.

Overall, we observed 17 errors in low-fidelity conditions compared to 14 errors in the high-fidelity conditions. There were no differences between the number of mild or severe errors. By contrast, we identified five moderate errors in the low-fidelity condition and two in the high (Figure 4 and Table 5).

## 5. Discussion

### 5.1. Principal Findings

While we saw some variation in the number and type of errors as a function of test fidelity and ecological validity, the difference was relatively modest (i.e., three additional errors in low-fidelity conditions) and inconsistent across severity levels. We observed more moderate errors in the low-fidelity condition but the same number of severe errors in both conditions. Overall, we observed the same error types in both conditions; increasing ecological validity did not improve our ability to detect specific types of usability issues. Nonetheless, given the small number of participants, scenarios, and observations, we hesitate to generalize to other technology evaluations or make broad testing recommendations. Instead, we believe there is a need for more comparative studies to identify subtle or specific effects of ecological validity on performance.

We propose several hypotheses to explain why participants made similar errors in both testing conditions. First, technology display screenshots (low-fidelity) looked like the live monitor (high-fidelity). Since the display did not include interactive features or affordances, the user experience may have been the same. Second, downstream participant behaviors were primarily cognitive (i.e., interpreting a display). There may have been insufficient task depth or breadth to see more cascading errors in workflow or ripple effects in complex adaptive systems. We might have seen more severe errors if we required participants to act on the data by adjusting medications or communicating with other clinicians. Finally, low-fidelity conditions may create a kind of “interference effect.” The artificial conditions of low-fidelity mock-ups and the absence of contextual cues in a laboratory setting may create interface usability issues—for example, the inability to recognize system status—or limit a participant’s situational awareness and response time.

### 5.2. Comparison to the Literature and Implications of Findings

While we did not see more errors or usability issues in the high-fidelity test conditions, we believe the interaction between test fidelity and error detection is complex. Increasing the fidelity of each dimension may not invariably increase test sensitivity or the ability to identify potential errors [40]. However, we cannot conclude that low-fidelity tests are always better or more sensitive than high-fidelity tests. On the contrary, it is still possible that high-fidelity simulations may identify significant and potentially severe usability issues that are complex, context-dependent, and otherwise hidden during tests with low ecological validity. Thus, low-fidelity usability tests should not be used in lieu of high-fidelity evaluations. Instead, they should be used earlier and potentially more frequently throughout the development and testing lifecycle. When selecting test fidelity, we believe the deciding factors to consider include the technology features of interest, the anticipated interaction between technology and the environment characteristics [40], the most important user goals, and the context of use. Nevertheless, this study’s findings suggest that low-fidelity tests are an efficient and cost-effective way to identify and mitigate many issues impacting ease of use, effectiveness, and safety. These findings also align with recommendations from leading usability experts to test early and often [41,42,43]. High-fidelity tests may offer deep insights into future implementation challenges, but we should also embrace “discount” testing to operate and innovate at the pace of healthcare.

There are four unresolved issues that demand further study. First, if ecological validity can influence the sensitivity of tests to detect usability errors, we must know where to apply our efforts. In resource-constrained settings, how do usability professionals predict what level of fidelity is needed to identify all important errors? Perhaps only some test dimensions must be high-fidelity to meet testing goals. Second, when there are so many dimensions of ecological validity—and within each dimension, so many levels of fidelity—professionals need a framework to know (1) which dimensions are associated with specific error types and (2) what fidelity level is sufficient to test a product’s performance thresholds. A starting point for developing this framework could be Kushniruk and Turner’s User–Task–Context matrix, which lists three dimensions relevant to HIT design and example attributes for each dimension [44]. Attempts to build a similar model for each of van Berkel’s seven dimensions would be more challenging. An ideal product would list the relevant attributes for each dimension and suggest strategies for creating low and high-fidelity versions. Third, we must know if there are certain dimensions that should always be high-fidelity. We presume testing participants should always possess knowledge of the clinical subject matter and use context. However, there may be other dimensions critical to safety or other key performance measures. Fourth, when designing tests with fidelity in mind, how low can you go? Jensen and colleagues proposed that extremely low-fidelity tests without a priori scenarios, functional prototypes, or patient data still provide valuable information [45]. These tests can foster discussion when identifying technology requirements, edge cases, or implicit user knowledge about the use context.

To address these issues, usability professionals should work towards a consensus on the minimum number of dimensions and attributes to consider when designing tests. One strategy that usability professionals can use when designing and reporting usability studies is to leverage—and expand—existing frameworks and guidelines [46,47,48]. Standardized reporting would enable researchers to strategically close gaps in our understanding of ecological validity and the effect specific dimensions have on the accuracy of findings.

### 5.3. Strengths and Limitations

There are several strengths of this work that deserve mention. First, we believe this is one of the first studies to use van Berkel’s theoretical framework as a scaffolding to build a usability testing protocol. In doing so, we are building the empirical database to explain how decisions of ecological validity influence usability testing findings, technology design decisions, and implementation outcomes. We also provide an extensible model to guide future testing in this arena. Second, this is one of the first studies to compare the effect of test fidelity in multiple dimensions (i.e., environment, scenario breadth, user behavior, patient involvement, and software). We compared two modalities of ecological validity in the lab, whereas most published reports compare the laboratory to the real world [24,25,26,29,30]. Third, we incorporated a HIT risk analysis into our protocol to develop a pre-identified list of errors. This increased the precision of our “testing forecast” and the instrumentation to search for these issues. At the same time, we could still identify and classify new error types.

There are also important study limitations affecting the explanatory power and generalizability of our results. First, we did not conduct comparative tests across all seven dimensions of van Berkel’s model. We could have included levels for user roles, user training, scenario urgency, and scenario typicality. Second, we configured fidelity at only two levels when, in fact, every dimension of fidelity exists on a continuum. For example, for patient involvement, our high-fidelity condition included a mannequin. This could have been a “mid-fidelity” condition, and we could have also included a standardized patient or an actual patient for the “high-fidelity” condition. As we noted above, the static screenshots of the interface look very similar to the actual device. The similarity in exposures may have caused a Type II (i.e., false negative) error. Third, we did not conduct naturalistic testing. Healthcare systems are complex adaptive systems with emergent properties, changing actors, and widely distributed workflow and cognition. This makes it extremely difficult to know what other usability issues went undetected. Fourth, we only tested one HIT. It would be informative to know how the technology, target user, and context-of-use interact with fidelity and testing outcomes. Fifth, we recruited 30 users—and only 15 per role. We do not know if this was the correct number to surface all usability issues. Deciding on the correct power for summative usability testing is a hotly debated topic in the literature [49]. While it has been argued that as few as 5 participants can identify over 80% of usability issues, research has shown that many more participants may be needed depending on the heterogeneity of users, the complexity of the product, and the goals of testing (e.g., formative testing for iterative re-design or summative testing for user acceptance) [50,51,52]. However, our sample size is in line with recommendations for summative evaluations of medical devices [53]. Sixth, there was a risk of contamination between dimensions or the order of exposure. For example, the onscreen data may have improved situational awareness, promoted new behaviors like cross-checking signals, and thereby decreased “high ANI misunderstanding” errors (#6).

## 6. Conclusions

More ecological validity does not always seem to be better when evaluating the usability of HIT. Our results suggest that high ecological validity does not consistently provide more information about the quality and defects of HITs. Low-fidelity testing can be an effective and cost-effective way of identifying and mitigating many problems associated with ease of use, efficiency, and safety. However, we do not recommend replacing all high-fidelity testing with low-fidelity testing. Instead, low-fidelity testing can be used early and more often so that usability researchers can better anticipate problems and guide development and implementation teams at the pace of healthcare.

## Figures and Tables

**Table 1:** List of use error types and severity. Mild = no injury, no patient discomfort; moderate = light patient discomfort; severe = serious injury or death.

Type No.	Error Type	Description of the Error	Level of Severity
1	ANI-ECG * confusion	Participant confuses ECG with ANI	Mild
2	No detection of overdosage	The participant does not recognize when an ANIm value over 80 for an unconscious patient represents a medication overdose	Moderate
3	Focus on ANI	The participant only uses the ANI index and neglects other data sources to evaluate the patient’s discomfort level	Moderate
4	Considering poor-quality data	The participant does not consider the quality of signal acquisition and bases her/his decision on poor-quality data	Moderate
5	Considering out-of-date data	The participant does not reset the ECG signal and bases her/his decision on out-of-date or erroneous data	Moderate
6	High ANI misunderstanding	The participant erroneously interprets the meaning of a high ANI on the screen	Severe
7	Low ANI misunderstanding	The participant erroneously interprets the meaning of a low ANI on the screen	Severe
8	Considering other patient data	The participant does not reset the values from the previous patient and bases her/his decisions on erroneous data	Severe

* ANI = analgesia nociception index; ECG = electrocardiogram.

**Table 2:** Description of the test environment according to the level of fidelity.

Ecological Validity Dimension	The Low-Fidelity Condition	The High-Fidelity Condition
1. User roles	Physicians and nurses specialized in intensive care. Participants had a minimum of two months of experience in intensive care and received training on the pain monitor two days before the test.	
2. Environment	Administrative room without other types of medical equipment and devices.	Simulated resuscitation rooms were similar to actual resuscitation rooms. The room was equipped with furniture and real medical technology and devices (infusion pumps, ECG * monitor, etc.). The simulation space mimicked a real resuscitation room in terms of temperature, ambient sounds, interruptive alarms, and disinfectant smell.
3. User training	All participants attended a training session at least two days before the test. This matches current training protocols with new equipment.	
4a. Scenarios, breadth	We did not set off monitor alarms to interrupt the participants.	Interrupting alarms from the monitors interrupted the participants, just like in real-life resuscitation rooms.
4b. Scenarios, depth	Five goal-based scenarios to test all eight identified use errors. Each scenario was performed twice, once in each condition. We furnished a summary of the patient’s case, including a description of the patient (e.g., age, gender, conditions), the clinical course, and a list of medications taken.	
4c. Scenarios, behavior	Participants were asked to verbalize how they would respond and what actions they would take.	Participants were asked to act on the mannequin as they would in real life.
5. Patient involvement	We did not include a patient or representation (i.e., a mannequin). Instead, the test moderator described the patient’s status. We provided screenshots of the patient parameters required for medical decision-making.	We used a mannequin capable of reproducing physiologically realistic reactions of the human body.
6. Hardware	Participants are shown screenshots printed on paper and a video on a computer screen with no possibility of interaction with the computer.	The ANI * pain monitor is an interactive screen framed by a plastic shell. Users can interact by directly pressing the interactive buttons on the screen. Depending on which buttons are pressed, parameters are modified, or windows are opened on the interface.
7. Software	The test was primarily performed using screenshots of the pain monitor. Scenario 4 (testing error 8) required the participant to see blinking ANI curves; thus, a video of the blinking screen was shown instead of a screenshot. Participants could see screenshots of data typically rendered on ECG, respiratory, and ANI pain monitors.	The test was performed with an actual ANI pain monitor. Participants could see the patient’s data typically rendered on ECG, respiratory, and ANI pain monitors in a live environment.

* ANI = analgesia nociception index; ECG = electrocardiogram.

**Table 3:** Demographic characteristics of the participants.

Profile	Number (Females; Males)	Mean Age in Years (*SD*)
Anesthesiologists	15 (9; 6)	28.26 (2.54)
Nurses	15 (9; 6)	31.93 (6.14)
Total	30 (18; 12)	30.01 (5.05)

**Table 4:** List of use errors, severity, and the number (percentage) of participants who committed them.

Type No	Error Type	Description of the Error	Severity Level	Low- Fidelity	High- Fidelity
1	ANI *-ECG * confusion	Participant confuses ECG with ANI	Mild	0 (0%)	0 (0%)
2	No detection of overdosage	The participant does not recognize when an ANIm value over 80 for an unconscious patient represents a medication overdose	Moderate	4 (13%)	2 (7%)
3	Focus on ANI	The participant only uses the ANI index and neglects other data sources to evaluate the patient’s discomfort level	Moderate	0 (0%)	0 (0%)
4	Considering poor-quality data	The participant does not consider the quality of signal acquisition and bases her/his decision on poor-quality data	Moderate	1 (3%)	0 (0%)
5	Considering out-of-date data	The participant does not reset the ECG signal and bases her/his decision on out-of-date or erroneous data	Moderate	0 (0%)	0 (0%)
6	High ANI misunderstanding	The participant erroneously interprets the meaning of a high ANI on the screen	Severe	3 (10%)	1 (3%)
7	Low ANI misunderstanding	The participant erroneously interprets the meaning of a low ANI on the screen	Severe	6 (20%)	7 (23%)
8	Considering other patient data	The participant does not reset the values from the previous patient and bases her/his decisions on erroneous data	Severe	3 (10%)	4 (13%)

* ANI = analgesia nociception index; ECG = electrocardiogram.

**Table 5:** Number of errors committed in each condition according to their severity.

Error Severity	Low-Fidelity	High-Fidelity
Mild (1 possible error type)	0	0
Moderate (4 possible error types)	5	2
Severe (3 possible error types)	12	12
Total (8 possible error types)	17	14

## Data Availability

Data is available on demand.
